# Assessing the Efficacy of the ARMOR Tool–Based Deprescribing Intervention for Fall Risk Reduction in Older Patients Taking Fall Risk–Increasing Drugs (DeFRID Trial): Protocol for a Randomized Controlled Trial

**DOI:** 10.2196/55638

**Published:** 2024-06-11

**Authors:** Rekha Priyadarshini, Madhavi Eerike, Sakthivadivel Varatharajan, Gomathi Ramaswamy, Gerard Marshall Raj, Jerin Jose Cherian, Priyadharsini Rajendran, Venugopalan Gunasekaran, Shailaja V Rao, Venu Gopala Rao Konda

**Affiliations:** 1 Department of Pharmacology, All India Institute of Medical Sciences Bibinagar Hyderabad India; 2 Department of General Medicine, All India Institute of Medical Sciences Bibinagar Hyderabad India; 3 Department of Community Medicine and Family Medicine, All India Institute of Medical Sciences Bibinagar Hyderabad India; 4 Division of Development Research, Indian Council of Medical Research Head Quarters New Delhi India; 5 Department of Pharmacology, Jawaharlal Institute of Postgraduate Medical Education & Research Pondicherry India; 6 Department of Geriatric Medicine, Jawaharlal Institute of Postgraduate Medical Education and Research Puducherry India; 7 Government Medical College Aurangabad India; 8 Department of Pharmacology, TRR Institute of Medical Sciences Hyderabad India

**Keywords:** deprescribing, geriatric, fall risk–increasing drugs, FRIDs, ARMOR tool, Assess, Review, Minimize, Optimize, and Reassess, falls, older patients, fall risk

## Abstract

**Background:**

Falls in older patients can lead to serious health complications and increased health care costs. Fall risk–increasing drugs (FRIDs) are a group of drugs that may induce falls or increase the tendency to fall (ie, fall risk). Deprescribing is the process of withdrawal from an inappropriate medication, supervised by a health care professional, with the goal of managing polypharmacy and improving outcomes.

**Objective:**

This study aims to assess the effectiveness of a deprescribing intervention based on the Assess, Review, Minimize, Optimize, and Reassess (ARMOR) tool in reducing the risk of falls in older patients and evaluate the cost-effectiveness of deprescribing FRIDs.

**Methods:**

This is an open-label, parallel-group randomized controlled academic trial. Individuals aged 60-80 years who are currently taking 5 or more prescribed drugs, including at least 1 FRID, will be recruited. Demographic data, medical conditions, medication lists, orthostatic hypotension, and fall history details will be collected. Fall concern will be assessed using the Fall Efficacy Scale, and fall risk will be assessed by the Timed Up and Go test and Tinetti Performance-Oriented Mobility Assessment tool. In this study, all treating physicians will be randomized using a stratified randomization method based on seniority. Randomized physicians will do deprescribing with the ARMOR tool for patients on FRIDs. Participants will maintain diaries, and monthly phone follow-ups will be undertaken to monitor falls and adverse events. Physical assessments will be performed to evaluate fall risk every 3 months for a year. The rationality of prescription drugs will be evaluated using the World Health Organization’s core indicators.

**Results:**

The study received a grant from the Indian Council of Medical Research–Safe and Rational Use of Medicine in October 2023. The study is scheduled to commence in April 2024 and conclude by 2026. Efficacy will be measured by fall frequency and changes in fall risk scores. Cost-effectiveness analysis will also include the incremental cost-effectiveness ratio calculation. Adverse events related to deprescription will be recorded.

**Conclusions:**

This trial will provide essential insights into the efficacy of the ARMOR tool in reducing falls among the geriatric population who are taking FRIDs. Additionally, it will provide valuable information on the cost-effectiveness of deprescribing practices, offering significant implications for improving the well-being of older patients and optimizing health care resource allocation. The findings from this study will be pertinent for health care professionals, policy makers, and researchers focused on geriatric care and fall prevention strategies.

**Trial Registration:**

Clinical Trials Registry – India CTRI/2023/12/060516; https://ctri.nic.in/Clinicaltrials/pubview2.php

**International Registered Report Identifier (IRRID):**

PRR1-10.2196/55638

## Introduction

With the growing burden of multimorbidity among the older population, the concurrent use of multiple medications, known as polypharmacy, has become increasingly common. Unfortunately, polypharmacy is often associated with a high treatment burden and a diminished quality of life; more alarmingly, it can lead to the use of potentially inappropriate medications, increasing the risk of falls. Fall risk–increasing drugs (FRIDs) encompass a group of medications known to induce falls or elevate the risk of falling. These FRIDs include psychotropic drugs (eg, antipsychotics, antidepressants, benzodiazepines, and sedative-hypnotics), cardiovascular agents (eg, loop diuretics, digoxin, antihypertensives, and antiarrhythmics), and other agents (eg, hypoglycemic drugs, nonsteroidal anti-inflammatory drugs, opioids, anticholinergics, and antihistamines) [1-3]. Notably, falls are the leading cause of hospital admissions in the older population, with a reported prevalence of falls due to FRIDs reaching 41% [4].

In response to these challenges, deprescribing has emerged as a crucial strategy. Deprescribing is defined as “the process of identifying and discontinuing medications in which existing or potential harms outweigh potential benefits within the context of an individual patient’s care goals, function, values, and preferences” [5]. Various deprescribing strategies have been explored to reduce adverse events, including the STOPP/START criteria, Beer’s criteria, the Medication Appropriateness Index, and the ARMOR (Assess, Review, Minimize, Optimize, Reassess) tool [6,7].

This study is designed to evaluate the effectiveness of the ARMOR tool in reducing the fall risk in the geriatric population with FRID prescriptions as well as the cost-effectiveness of this intervention.

## Methods

### Study Design and Setting

This is a multicenter, open-label, parallel-group randomized controlled study. This non–drug intervention academic trial will be conducted at selected sites after obtaining approval from all the respective Institutional Ethics Committees (IECs). The trial application has been submitted for registration with the Clinical Trial Registry – India, prospectively. The study duration is approximately 2 years, and the patient participation duration in the study is approximately 1 year**.**

### Participants

Participants will be enrolled in the study over a period of 1 year (approximately) based on the study’s eligibility criteria. Participants aged between 60 and 80 years; currently prescribed 5 or more medications, including at least one FRID; with a Montreal Cognitive Assessment (MoCA) score above 26; capable of walking 10 meters either independently or with the assistance of a walking aid (eg, walking stick, canes, or calipers); and voluntarily willing to participate and provide informed consent will be enrolled into the study.

Critically ill patients, patients with known neurological disorders (eg, cognitive impairment, dementia, Parkinsonism, or a history of stroke), older individuals receiving home care or specialized care, and older adults relying on a walker or wheelchair for mobility will be excluded from the study**.**

### Sampling Technique

This study will follow a proportionate-to-size sampling technique. Initially, the participants will be recruited from 4 major departments where FRIDs are most commonly prescribed. The departments will be sequentially numbered as 1, 2, 3, and 4 based on the proportion of older patients attending the department. The proportion of recruitment will be 50%, 20%, 15%, and 15% from departments 1, 2, 3, and 4, respectively. In the first round, the first department will be visited first for participant recruitment, and in the second round, the second department will be visited first, and so on for further rounds. Once the required number of participants is recruited from that outpatient department, that department will be removed from the sequential list. On the day of the visit to the respective department, eligible participants who are willing to participate in the study will be included, with 1 patient recruited per day.

### Randomization

#### Prescriber or Clinician randomization

After obtaining written informed consent, all treating physicians will be randomized using a stratified randomization method based on seniority in our study design. The clinicians will be divided into strata based on their seniority levels, for example, junior resident, senior residents, mid-level doctors (eg, assistant and associate professor levels), and senior doctors (eg, additional professor and professor cadre level). They will then randomly assign participants within each stratum to the intervention or control group. This stratified randomization ensures an equal distribution of doctors across the study groups, minimizing the potential for bias and contamination. Additionally, it accounts for any potential variations in expertise, experience, or treatment preferences among doctors of different seniority levels. The random allocation sequence will be generated using computer-generated random numbers. Enrolled participants will then be treated by the clinician randomized to the respective group.

#### Blinding (Masking)

Considering the nature of the intervention proposed, this study is designed as an open-label study; the participants and the intervention implementing team will not be blinded to the intervention. However, the statistician performing data analyses will be blinded to group assignments.

#### Baseline Assessment

Demographic details, such as age, gender, occupation, education, BMI, disease conditions details, and list of drugs used (including FRIDs with their costs and orthostatic hypotension details) will be collected from all the participants. Drug details, such as the name of the drug, indication, frequency, route of administration, and duration of use will also be collected. The rationality of the prescriptions will be assessed by the World health Organization prescription indicators.

Participants will then be asked about their previous fall history using the fall history questionnaire of McCrum 2020 [8] and their concern for falls assessed by the Fall Efficacy Scale [9,10]. Quality of life will be assessed by administering the EQ-5D-5 L questionnaire. Once the baseline data collection is completed, they will be assessed for fall risk. The fall risk assessment will be conducted by a staff nurse using the Timed Up and Go test and the Tinetti Performance-Oriented Mobility Assessment Tool.

### Intervention Details

Clinical pharmacologists will do steps 1 and 2 of the ARMOR tool for the recruited participants’ prescriptions and prepare a summary report of all the recruited participants’ prescriptions.

### Test Group

Participants randomized to this group will be managed using the ARMOR tool–based deprescribing intervention, which contains 5 steps—Assess, Review, Minimize, Optimize, and Reassess. A clinical pharmacologist provides a summary report of steps 1 and 2 of the ARMOR tool to the treating physician. The treating physician will complete steps 3 to 5.

### The ARMOR tool

The ARMOR tool [11] is designed to guide health care professionals in a structured approach to medication management, emphasizing the importance of regular assessment, review, modification, optimization, and reassessment. It promotes patient-centered care and aims to enhance medication safety and effectiveness.

#### Step 1: Assessment

A comprehensive assessment of the patient's medication regimen will be done during this step.

#### Step 2: Review

In this step, the pharmacologist along with the treating physician will review the assessment findings (ie, the overall effectiveness and necessity of each medication) and will identify any potential risks or harms associated with continued use.

#### Step 3: Modify

Based on the review submitted, the clinician may consider a deprescribing intervention for medications that are no longer necessary, ineffective, or potentially harmful. If required clinicians will consider adjusting dosages, changing formulations, or switching to alternative medications.

#### Step 4: Optimize

In this step, clinicians will focus on enhancing the patient's medication regimen to achieve the best possible outcomes. They may initiate new medications when necessary, optimizing dosages for maximum efficacy, and addressing any identified gaps in therapy.

#### Step 5: Reassess

After modifications and optimization, the clinician will continue to monitor the patient's response to the medication changes. They reassess the patient's symptoms, side effects, and overall treatment outcomes.

### Control Group

Participants randomized to the usual care group will receive treatment as per the standard practice or protocol. As there are no established gold standards for effective, deprescribing interventions, usual care is chosen as a comparator.

### The Follow-Up Phase

Each participant will be followed for a period of 1 year after their enrollment into the study. Both groups will be followed up for 1 year. Each participant will be given a diary to record the falls and other adverse events. They will be followed up monthly over the phone for falls and for occurrence of other adverse events and for improving adherence to the intervention protocols of the study. Every 3 months, they will be followed up physically to assess fall risk, inquire about any fall history, and note any adverse events during that period.

### Unscheduled Visits

In case of any adverse events and serious adverse events during the study period, there may be an unscheduled visit planned for the concerned participant. The details of any such event will be recorded and appropriate treatment will be provided. When trial participants experience an injury, adverse event, or serious adverse events, they will be noted, and appropriate treatment will be given.

### End of Study

**Figure 1:**
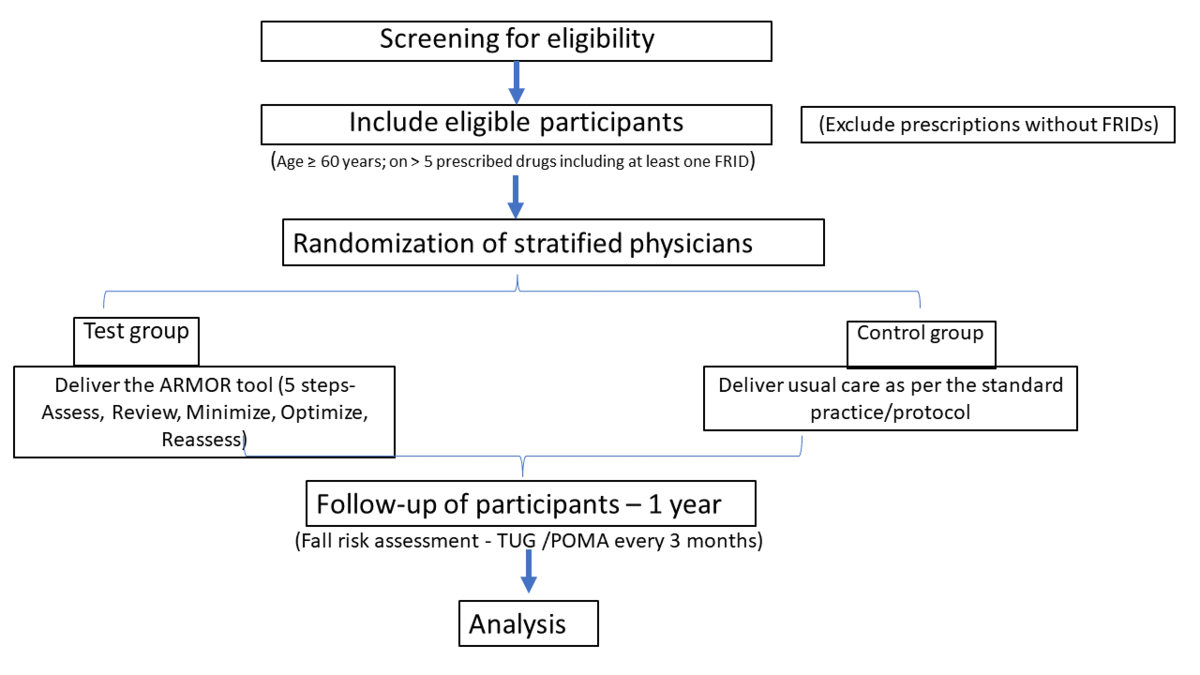
Flowchart of the overall study methodology. ARMOR: Assess, Review, Minimize, Optimize, and Reassess; FRID: fall risk–increasing drug; TUG: Timed Up and Go; POMA: Tinetti Performance-Oriented Mobility Assessment.

At the end of the study (after 1 year of follow-up), the complete information, such as the number of drugs with their details, fall history, fall concern, fall risk, and quality-adjusted life-year will be assessed. The rationality of the prescriptions will be assessed by the World Health Organization prescription indicators. Figure 1 provides an overview of the study methodology.

### Adverse Events or SAE

Adverse events will be recorded in the adverse event form. The details will be collected in the adverse drug reaction reporting form and causality assessment will be done to find the adverse event’s association with FRIDs. In the case of SAE, we will be recording and reporting the serious adverse events to regulatory authorities.

### Statistical Methods and Analysis

#### Sample Size

The sample size is estimated using the sample size formula for estimating proportion between two groups**.** Considering the reported fall incidence with FRIDs as 73% [12], the sample size is estimated to be 478 (each arm: 239), with 90% power (as the outcomes measures are only falls and do not include any mortality, we have set the power to 90%) and 5% level of significance to detect a minimum of 20% reduction in falls in the intervention group relative to the control group (relative risk reduction of 20%). Considering the dropout of 10%, the final sample size will be 526 (263 in each arm). A total of135 participants are expected to be recruited from each center.

#### Data Analysis

The data will be entered in Research Electronic Data Capture (REDCap) and will be analyzed using Stata (version 16; StataCorp) software. Data will be stored in a secure server or cloud-based platform. A principal investigator and a co–principal investigator will have access to the REDcap data. The statistician will be given access to the blinded REDcap data. The occurrence of falls and fall risk during the study period will be summarized as frequency and percentages. The number of falls at these time points will be reported using the median (IQR) values. The fall risk score will be reported as mean (SD) or median (IQR) based on the normality of the data. The Mann-Whitney *U* test and Friedman test (chi-square test) will be used to analyze the change in the frequency of falls (%) at each time point between the groups and within the groups, respectively. The chi-square test will be used to analyze the fall risk score category at each time point between the groups. Additionally, repeated measures ANOVA (or Friedman test) and unpaired *t* test (or Mann-Whitney *U* test) will be used to assess the within-group fall risk score at different time points and at each time point within the group, respectively. Poisson regression analysis (both bivariate and multivariate) will be conducted to explore the association between the dependent variable (ie, the number of falls and fall risks assessed using Timed Up and Go and the Performance-Oriented Mobility Assessment) and independent factors (eg, age, gender, education, chronic illness, and intervention category). Adjusted risk ratios with 95% CIs will be reported for the factors included in the multivariate Poisson regression model. The rationality of prescriptions will be summarized using frequencies and percentages. Subgroup analysis will be performed for participants with recurrent fall history and different age categories.

#### Incremental Cost-Effectiveness Ratio (ICER)

Incremental Cost-Effectiveness Ratio (ICER) will be calculated by dividing the difference in costs between the interventions by the difference in outcomes. The ICER represents the additional cost per unit of outcome achieved by one intervention compared to another.

We will be following the intention-to-treat strategy to analyze the data. Therefore, even participants who discontinue but have at least 1 follow-up will be included in the study to achieve the required sample size, taking into account their age and health status.

### Study Monitoring

This study will be monitored by the ICMR monitoring team. The investigator will allocate adequate time for such monitoring activities and ensure access to all the above-noted study-related documents and study-related facilities.

#### Data and Safety Monitoring Board

A Data and Safety Monitoring Board will be constituted for this study independent of the study team members. The team will look into the safety of the intervention proposed with an interim analysis report.

#### Interim Analyses

Two interim analyses will be conducted during the study period: the first after the completion of the first follow-up of 200 participants and the second after reaching 400 participants.

### Ethical Considerations

The study will be conducted as per the ICMR National Bioethical Guidelines 2017 [13] and New Drugs and Clinical Trial Rules 2019 [14]. The study received approval from All India Institute of Medical Sciences Bibinagar, Institutional Ethics Committee (AIIMS/BBN/IEC/JUNE/2023/294) on June 16, 2023.

Eligible participants who are willing to participate in the study will be enrolled after obtaining written informed consent.

Confidentiality of participation will be maintained, and personal identity will not be revealed during data collection, analysis, presentations, and publications. Study records will be preserved for 3 years and will be made available only to authorized persons conducting the study and to the funding agencies without revealing participant identities.

Reimbursement of Rs 500 (about US $6) per visit will be provided for participation in the study, as participants will be asked to visit the hospital every 3 months after their enrollment into the study.

For any foreseeable and unforeseeable complication, adequate compensation will be provided depending on the nature of the injury or illness. In addition, trial insurance coverage will be provided for any unforeseeable risks related to the study.

## Results

The study received a grant from the Indian Council of Medical Research–Safe and Rational Use of Medicines (ICMR–SRUM) in October 2023. The study is scheduled to start in June 2024 and conclude by 2026. The study protocol is registered with the Clinical Trial Registry – India (registration number CTRI/2023/12/060516). Data collection and follow-ups are expected to be completed by the third quarter of 2026. The results are expected to be submitted for publication in 2026. Based on the results of this study, modules will be prepared and workshops will be organized for the prescribers on deprescribing interventions. The research findings will be presented at the national/international conference for large-scale public dissemination. The results of this study will be published in indexed peer-reviewed scientific journals. The full protocol, participant-level data set, and statistical code will be made publicly available after the completion of the study.

## Discussion

### Expected Outcomes

Polypharmacy has become a major concern in the geriatric population considering their multimorbid conditions. The number of prescribed drugs tends to increase with age, especially among individuals with multiple comorbidities. Although these drugs are prescribed to treat various conditions, some of them, known as FRIDs, contribute to falls in older people. FRIDS include psychotropic drugs (eg, antipsychotics, antidepressants, benzodiazepines, and sedative-hypnotics), cardiovascular agents (eg, loop diuretics, digoxin, antihypertensives, and antiarrhythmics), and other agents (eg, hypoglycemic drugs, nonsteroidal anti-inflammatory drugs, opioids, anticholinergics, and antihistamines). Reports indicate that falls among the older population are significant causes of morbidity, with FRIDs identified as one of the contributing factors for falls.

Deprescription is defined as systematically removing the drug, reducing the dose, or changing the medication without affecting the patient’s health condition. There are various deprescribing tools available for controlling polypharmacy, but none has been considered as a standard with proven effectiveness, as they are often complicated, tedious, and not user friendly for implementation in the real-world settings.

The ARMOR tool-based deprescribing intervention, which contains 5 steps (Assess, Review, Minimize, Optimize, and Reassess) is very convenient and easy to use by physicians and will help in reducing the falls and fall risk in older people who use FRIDs. It is an effort to approach polypharmacy and inappropriate medications in a systematic and organized fashion. It is a functional and interactive evidence-based tool that considers a patient’s clinical profile and functional status with the primary goal of improving functional status and mobility. This tool also emphasizes quality of life as a key factor for making decisions on changing or discontinuing medications.

A study was conducted to evaluate the implementation of the ARMOR tool to address polypharmacy in a rural nursing care facility. This nonrandomized, single-arm, pre- and postbaseline intervention study design was conducted with 600 residents using the ARMOR tool with an interdisciplinary team as an intervention in 7 centers. The residents were on psychotropic medications, experiencing recent or frequent behavioral disturbances causing distress to themselves or others, and exhibiting significant changes in their medical condition [15]. The results showed a negative trend in pain, falls, and anti-anxiety medication use, along with positive results in terms of reduction in the use of psychotropic medications. The average rate of reduction or improvement in antipsychotic risk with the intervention was 13.9% [15].

In India, there are no randomized controlled studies available on the use of this functional and interactive evidence-based tool. This trial will provide essential insights into the efficacy of the ARMOR tool in reducing falls among the geriatric population taking FRIDs. Additionally, it will provide valuable information on the cost-effectiveness of deprescribing practices, offering significant implications for improving the well-being of older patients and optimizing health care resource allocation. The findings from this study will be pertinent for health care professionals, policy makers, and researchers specializing in geriatric care and fall prevention strategies.

### Conclusions

This randomized controlled trial will provide valuable insights into the potential of the ARMOR tool–based intervention to reduce the risk of falls in older patients and its cost-effectiveness in deprescribing FRIDs. The positive outcomes observed in this study would underscore the importance of medication review and optimization as part of comprehensive fall prevention strategies for the older population. Further, this research will guide the implementation of evidence-based interventions in clinical practice. This study marks the first of its kind to be conducted in India on deprescribing intervention using the ARMOR tool with a randomized control led design.

## Appendix

Multimedia Appendix 1 Peer-reviewer report from ICMR.

## Abbreviations

ARMOR: Assess, Review, Minimize, Optimize, Reassess
FRID: Fall Risk Increasing Drugs
ICMR: Indian Council of Medical Research
REDCap: Research Electronic Data Capture
