# Supplementary material for: Assessing the Efficacy of the ARMOR Tool–Based Deprescribing Intervention for Fall Risk Reduction in Older Patients Taking Fall Risk–Increasing Drugs (DeFRID Trial): Protocol for a Randomized Controlled Trial
Source: JMIR Res Protoc. 2024 Jun 11;13:e55638. doi: 10.2196/55638 (PMC11200035; doi:10.2196/55638)
Supplement: Multimedia Appendix 1 [file resprot_v13i1e55638_app1.pdf]

Gmail

madhavi Eerike <dr.madhavieerike@gmail.com>

Comments from experts

ICMR SRUM <icmr.srum@gmail.com> Thu, May 25, 2023 at 9:30 PM
To: dr madhavieerike <dr.madhavieerike@gmail.com>, Jerin Cherian <jerin.cherian.dhr@gmail.com>

Dear applicants to the SRUM Task Force

Greetings from ICMR!
Here by attaching the general comments as well as proposal specific comments received for your proposal during the 7th meeting of ICMR-SRUM NTF.

Discussion and general comments

The general comments which are applicable for all the shortlisted proposals are as follows:

- To include appropriate subject experts with necessary expertise in the project team especially in those proposals which have tool development as the major objective. Details of validation of the tool are not adequately mentioned in the proposals, however appropriate experts may be identified with the help of ICMR for conducting workshops for tool development and tool validation.
- All proposals will have to describe their data management plan in detail. It would be ideal to electronically capture data so that it will be easier for analysis, and reduce manpower requirements.
- Some proposals have not adequately described the significance of the assigned project topic in Global and Indian context. The applicants could not adequately justify the need for the project, for which a quick review of current global and Indian scenarios may be included, with special emphasis on how their projects can held bridge the gap. All proposals may consider including a tabular summary of current evidence, outline gaps, and explain how their studies can address these gaps. These may be linked to the periodic deliverables.
- Budget for travel should be reduced for most proposals unless absolutely necessary and a uniform policy for publication cost as well as travel will be made by the ICMR. There must be a uniform policy for publication support for all proposals. INR 1,50,000 can be provided for up to two manuscripts (budgeted for one manuscript in second year and third year respectively) that will have to be published in peer-reviewed, indexed journals with IF $\geq$ 5.
- Budget seems very high in most proposals, especially for travel, journal publications and human resources. There is a need to truncate the budget and rationalize against the proposed tasks. ICMR may discuss with the applicants to modify the budget suitably and ensure adequate justification.

Deprescribing interventions (ARMOR) to reduce risk of falls in Geriatric population on Fall Risk Increasing Drugs (FRIDs) – A Randomized Control Trial-Dr. Madhavi Eerike

Technical comments from reviewers

- Response to the comments from the reviewers were not captured clearly in the respective table. The reviewers suggested to applicant to kindly provide detailed responses to the comments in the tabular summary and refer to the modifications in the proposal wherever applicable.
- The reviewers had recommended to include necessary experts on the study team. Geriatric doctors have been proposed by the applicant, however the reviewers felt that this may be inadequate. The reviewers require the applicant to include multi disciplinary teams (such as nurses - the reasons provided for their non-inclusion was not satisfactory) and to substantiate the credentials of the team with respect to their specific roles in the study.
- The reviewers had earlier recommended the applicant to randomise the physicians who will obtain training for deprescribing (those who will be providing the intervention).
- Details of training given by clinical pharmacologist and pharmacist to physicians not provided in the proposal.
- The proposal does not clearly mention how the investigators will attribute the fall events to the FRIDS and not as a result of other reasons for fall. Since this is the primary objective of the study, there will

need to be suitable modifications made in the design to be able to identify falls as a result of FRIDS or otherwise.

- Reference selected for calculating sample size is not given in the proposal, especially the estimation of 20% reduction in falls. There needs to be ample justification of the a-priori estimates from literature.
- Feasibility of the intervention has not been described by the applicant. The reviewers considered this information critical before the proposal can be recommended. Similarly study design modifications to prevent contamination can not be satisfactorily achieved by randomization alone, as described by the applicant.
- The applicant proposes block randomisation, which may not be necessary for the study. Instead, stratified randomization (not more than 4) based on seniority should be considered in doctors.
- Blinding strategy needs to be clarified better in the proposal. Nurses from the same unit/ ward in the hospital may have access to critical treatment information that can interfere with the independence of the outcome measurement.
- A tabular summary of relevant literature needs to be given in the main proposal, besides what is in the annexure.
- Pragmatic concerns were raised by the reviewers over the enrollment to the study. Rather than appointing a clinical pharmacologist/ clinical pharmacist for enrollment, it may be ideal to utilise the treating physician for enrollment of patients to the study. Patients may otherwise be reluctant to enrol in a study that their treating physician does not invite them to. The applicant was requested to deliberate the concerns with their study team and identify suitable solutions.
- Planned subgroup analysis will need to be described a-priori
- The applicant has mentioned that they will obtain ethics approval, however, expected ethical issues of the study have not been identified. The consequences of deprescribing and ethical challenges need to be identified and addressed. ICMR's 12 principles may be referred to for this purpose.
- Recruitment strategy needs to be clearly identified.

Budget comments from reviewers

- The reviewers commented that there may not be a need for a project technical officer and a nursing staff as their roles and responsibilities seem to be overlapping (absence of clear justifications by the applicant).
- If required, one project technical officer may be recruited for the lead site for project coordination. These positions may be recruited only during months 10 to 35 in line with the gantt chart.
- Compensation under recurring costs are not valid, especially since there is an insurance cover for the trial.
- Budget containing insurance for the trial participants, lab tests and adverse event management could be removed/ revised. The intervention is not expected to have serious adverse events and the need for insurance, lab tests and adverse event management does not seem valid. Budget for data management can be increased.

Final recommendations from reviewers

Suitable modifications may be made by the applicant to incorporate the inputs from the TAG. The TAG recommended that the proposal may need significant modifications to outline all the steps involved in tool development and validation. The applicant will also need to describe in detail the need for such a tool and have more clarity on its potential applications. **In its current stage, the proposal is weak and the applicant may be given time to make suitable revisions. The proposal was recommended for further development and review by the TAG.** Necessary capacity building may be carried out by ICMR.

Kindly note that if we receive any further comments from the experts, we will soon inform you through mail. Kindly incorporate the necessary changes in your proposal as well as budget as soon as possible.

Regards
Sowparnika Treasa Sabu
Research Associate - III
Division of BMS
ICMR HQ
For Dr. Jerin Jose Cherian
Scientist D (Med), Division of BMS,
ICMR HQ.

PROTOCOL

Complete Title	**De**prescribing interventions (ARMOR) to reduce the risk of falls in geriatric population on **F**all **R**isk **I**ncreasing **D**rugs (FRIDs) – An open label, and parallel group randomized controlled trial
Short Title	DeFRID trial
Protocol Identification no.	AIIMS/BBN/ PHARM/SRUM/PROTOCOL/01
Principal Investigator	Dr.MadhaviEerike
Protocol Date and version	Version 04 dated 01.06.2023
Amendment 1 Date:	Amendment 3 Date:
Amendment 2 Date:	Amendment 4 Date:

Confidential

This document contains confidential information. It is to be used by investigators, consultants, regulatory authorities or ethics committees.
The information is not to be disclosed to other parties without prior written permission from ICMR except where required by applicable laws in India.

	**Comment**	**Response**	**Modification done in page no**
1.	To include appropriate subject experts with necessary expertise in the project team especially in those proposals which have tool development as the major objective. Details of validation of the tool are not adequately mentioned in the proposals; however appropriate experts may be identified with the help of ICMR for conducting workshops for tool development and tool validation.	Thank you for the valuable suggestion. Our study focuses on testing of ARMOR as deprescribing intervention. As suggested we are including appropriate subject experts to review and oversee various activities throughout the study period.	3
2.	All proposals will have to describe their data management plan in detail. It would be ideal to electronically capture data so that it will be easier for analysis, and reduce manpower requirements	Thank you for the comment regarding the data management plan. We included detailed description of data management plan in our protocol. As suggested we will try to employ the electronic data capture methods in our study.	31-33
3.	Some proposals have not adequately described the significance of the assigned project topic in Global and Indian context. The applicants could not adequately justify the need for the project, for which a quick review of current global and Indian scenarios may be included, with special emphasis on how their projects can held bridge the gap. All proposals may consider including a tabular summary of current evidence, outline gaps, and explain how their studies can address these gaps. These may be linked to the periodic deliverables.	Thank you for the comment. We have included comprehensive justification for the need of our project, including a review of current global and Indian scenario.we are including the tabular summary of current evidence in our proposal.	12-13 - Table as annexure
4.	Budget for travel should be reduced for most proposals unless absolutely necessary and a uniform policy for publication cost as well as travel will be made by the ICMR. There must be a uniform policy for publication support for all proposals. INR 1,50,000 can be provided for up to two manuscripts (budgeted for one manuscript in second year and third year respectively)	Thank you for your feedback regarding the budget allocation for travel and publication costs in our proposal. We revised our budget allocation and adhere to the guidelines set by the ICMR for travel and publication of manuscript.	42-47

	that will have to be published in peer-reviewed, indexed journals with IF > 5.		
5.	Budget seems very high in most proposals, especially for travel, journal publications and human resources. There is a need to truncate the budget and rationalize against the proposed tasks. ICMR may discuss with the applicants to modify the budget suitably and ensure adequate justification	Thank you for your valuable suggestion.We revised our budget to align with the proposed tasks and optimize the utilization of resources.	52-57

Technical comments

**Sno**	**Reviewer comment**	**Response**	**Page No**
1.	Response to the comments from the reviewers was not captured clearly in the respective table. The reviewers suggested to applicant to kindly provide detailed responses to the comments in the tabular summary and refer to the modifications in the proposal wherever applicable	Thank you for your feedback regarding the clarity of capturing the responses to the reviewers' comments in the respective table. We appreciate your suggestion to provide detailed responses within the tabular summary and include page numbers to refer to the modifications made in the proposal where applicable.	-
2.	The reviewers had recommended including necessary experts on the study team. Geriatric doctors have been proposed by the applicant, however the reviewers felt that this may be inadequate. The reviewers require the applicant to include multi disciplinary teams (such as nurses - the reasons provided for their non-inclusion was not satisfactory) and to substantiate the credentials of the team with respect to their specific roles in the study	Thank you for your suggestion to include an expert team from various disciplines. The composition of our expert team now includes individuals with backgrounds in Geriatrics, General medicine, PMR, Clinical Pharmacist and nursing officer.<br>Nursing officers are recruited as project staff in this project.<br>Please note the justification for non inclusion of nurses was given for Phase I study (KAP study) and not for RCT – phase II study**.**	3
3.	The reviewers had earlier recommended the applicant to randomize the physicians who will obtain training for deprescribing (those who will be	Thank you for the suggestion. Initially we proposed block randomization for treating clinicians, later as per the suggestions of reviewers (technical comment-8) we have modified this to stratified randomization based	29-30

	providing the intervention).	on seniority. The details are included in the protocol.	
4.	Details of training given by clinical pharmacologist and pharmacist to physicians not provided in the proposal	Thank you for the suggestion. The step wise approach will be followed to train physicians.<br>At first step Pharmacologists will deliver lectures or presentations on deprescribing intervention principles, various tools used during this intervention including ARMOR, medication review techniquesand evidence-based guidelines.<br>Later we are planning to conduct one workshop with case scenarios where physicians can practice deprescribing techniques in a safe and controlled environment. And during the conduct of the study, we will engage physicians in case-based discussions to promote critical thinking and decision-making skills in deprescribing scenarios. And small group discussions to facilitate small-group discussions /regular meeting with physicians where they can share their experiences, challenges, and success stories related to deprescribing.<br>Last option will be preparing the E-Learning Module which will cover various deprescribing topics, including specific medication classes, patient-centered communication, tools for deprescribing, and monitoring strategies. These modules can be used by physicians at their convenient time	47-48
5.	The proposal does not clearly mention how the investigators will attribute the fall events to the FRIDS and not as a result of other reasons for fall. Since this is the primary objective of the study, there will need to be suitable modifications made in the design to be able to identify falls as a result of FRIDS or otherwise	Thank you or the comment.<br>Due to the multifactorial nature of falls it will be a challenge for us to identify falls solely due to FRIDs. However we will utilize the following strategies to enhance the identification process:<br>**Baseline assessment**: Collect comprehensive baseline data on participants, including their medical history, medications, and fall risk factors. This will help establish a foundation for assessing changes in fall rates during the trial and identifying potential contributors to falls.<br>We included adverse event reporting system where participants and healthcare providers can report falls and other related incidents. We will also follow up over phone to enquire about falls and other adverse events. Participant will be	25 ,AE-27

		given dairy to record any adverse events including falls happening during the study period. We will also encourage participants to and report any falls promptly.<br>We will conduct a comprehensive assessment for each reported falls through fall history questionnaire and fall efficacy scales. This will capture the review of circumstances, location, time, potential causes, and participant characteristics. This assessment can help identify any factors specifically related to FRIDs, such as the timing of medication administration and the presence of known side effects.<br>Fall events will be reviewed through multidisciplinary team for evaluating the potential contribution of FRIDs.<br>**Monitoring and documentation**: we will implement monitoring and documentation procedures throughout the trial to capture participant adherence to FRID withdrawal or continuation protocols. This information will provide insights into the relationship between FRID use and fall events.<br>.	
6.	Reference selected for calculating sample size is not given in the proposal, especially the estimation of 20% reduction in falls. There needs to be ample justification of the a-priori estimates from literature	Thank you for the comment. The article from Van der valde et al reported 20% absolute risk reduction in the FRIDS group compared to non FRIDS group. Since we couldn't find any articles from India we considered the 20% absolute risk reduction in calculation of sample size. The article has already been added as reference in the sample size calculation section.<br>**Ref:***van der Velde N, Stricker BH, Pols HA, van der Cammen TJ. Risk of falls after withdrawal of fall-risk-increasing drugs: a prospective cohort study. Br J Clin Pharmacol. 2007 Feb;63(2):232-7*	40
7.	7a. Feasibility of the intervention has not been described by the applicant. The reviewers considered this information critical before the proposal can be recommended.	Thank you for valuable comment.<br>The present is planned as open label study. It is well known that conducting a clinical trial without blinding poses certain challenges, such as the potential for bias and the influence of patient & clinician expectations on treatment outcomes. However, we believe that with careful planning and implementation, the feasibility of the deprescribing intervention is possible.	26-27

		We will establish a strong clinical rationale for the deprescribing intervention. This includes reviewing existing evidence, guidelines, and expert opinions supporting the effectiveness and safety of the deprescribing approach in the target population. A solid clinical foundation provides a strong basis for the feasibility and potential benefits of the intervention.<br>Detailed procedure for deprescribing intervention is included in the study protocol and the same will be explained to treating doctors such as approach for deprescribing, including the specific criteria for medication reduction or discontinuation. Participating clinicians receive appropriate training and education on the deprescribing intervention. The training will focus on the rationale behind deprescribing, how to use proposed deprescribing tool, patient selection criteria, and monitoring for potential adverse events or withdrawal effects.<br>We will educate patients/caregivers about the deprescribing intervention, its potential benefits, and the importance of their active participation. We will engage patients in shared decision-making processes, involving them in discussions about medication reduction or discontinuation. By providing comprehensive information and addressing patient concerns, feasibility can be improved as patients become more willing to participate in the deprescribing intervention.<br>Foster collaboration, regular meetings and feedback sessions both from treating clinicians and patients will reduce feasibility concerns.	
	7b. Similarly study design modifications to prevent contamination cannot be satisfactorily achieved by randomization alone, as described by the applicant.	Thank you for the comments. Apart from stratified randomization the additional measures that will be followed during the study period to avoid contamination<br>1. We will give training session to treating clinicians at the beginning of the study period. During training session we will ensure that they have a clear understanding of the trial objectives, their role in the study, and the importance of maintaining group independence.We will emphasize the significance of adhering to the assigned treatment strategies and avoiding	30-31

		discussions or sharing of information between groups.<br>2.We will conduct regular meetings or updates with doctors in each group to reinforce the trial objectives and get clarifications if they have concerns or questions<br>3. Regular monitoring will be done to identify any potential contamination by periodic reviews of patient records, observations of clinical practices, and assessments of adherence to the assigned treatment strategies<br>4. While educating patients/care givers we will emphasize the need to only discuss their problems/care with their assigned doctors and not to share information between group and also to encourage patients/care givers to report any instances where they feel information or treatments may have been exchanged between doctors	
8.	The applicant proposes block randomization, which may not be necessary for the study. Instead, stratified randomization (not more than 4) based on seniority should be considered in doctors	Thank you for the suggestion.<br>Block randomization is proposed initially to ensure balanced allocation and prevent contamination. We agree that stratified randomization based on seniority could be an alternative approach that may further enhance the independence and integrity of the trial.<br>Accordingly, we revised our randomization plan and incorporate stratified randomization based on seniority into our study design.<br>This involve dividing the doctors into strata based on their seniority levels (e.g., junior doctors, senior residents, mid-level doctors (assistant and associate professor level), senior doctors (additional and professor level), and then randomly assigning participants within each stratum to the intervention or control group. This ensures an equal distribution of doctors across the study groups, minimizing the potential for bias and contamination. It will also account for any potential variations in expertise, experience, or treatment preferences among doctors of different seniority levels.	29-30
9.	Blinding strategy needs to be clarified better in the proposal. Nurses from the same unit/ ward	Thank you for the comment. To minimize the influence of nurses with access to treatment information, we will implement the following	32

	in the hospital may have access to critical treatment information that can interfere with the independence of the outcome measurement.	measures:<br>Allocation concealment will be implemented using sealed envelopes to ensure that the allocation sequence is concealed from the nurses performing outcome measurements. We will provide training and education to the nurses involved in the outcome assessments. This training will emphasize the importance to maintain independence in measurements, and the potential biases that can arise from having knowledge of the treatment allocation. By increasing awareness and understanding, we aim to minimize unintentional biases and ensure consistent and reliable outcome measurements.	
10.	A tabular summary of relevant literature needs to be given in the main proposal, besides what is in the annexure	We appreciate the suggestion to include a tabular summary of relevant literature in the main proposal.The tabular summary includes the following elements- study reference, objectives, sample size, study population, intervention details and main findings ofimportant outcomes	22-23 separate table
11.	Pragmatic concerns were raised by the reviewers over the enrollment to the study. Rather than appointing a clinical pharmacologist/ clinical pharmacist for enrollment, it may be ideal to utilize the treating physician for enrollment of patients to the study. Patients may otherwise be reluctant to enrol in a study that their treating physician does not invite them to. The applicant was requested to deliberate the concerns with their study team and identify suitable solutions.	Thank you for reviewers' feedback and their pragmatic concerns regarding the enrollment process for the study. As suggested we will utilize treating physicians during enrollment phase.<br>To ensure active involvement of treating physicians in the enrollment process, we will establish effective communication and collaboration channels with them and will be training about the study objectives, and the deprescribing intervention to the treating physicians, highlighting the potential benefits to their patients and emphasize the importance of their role in identifying eligible participants and inviting them to participate in the study.	28
12.	Planned subgroup analysis will need to be described a-priori	Thank you for the inputs. We will be doing confirmatory subgroup analysis for the following sub groups to assess the effect of intervention on the frequency of falls.<br>1) The following age categories will be used for subgroup analysis. Category 1: 65-74 years Category 2: 75 – 80. The deprescribing intervention studies utilized similar age categories for testing its efficacy.<br>2) It has been reported that deprescribing (discontinuation or dose reduction) of FRIDs in older	39

		with fall history is an effective single intervention for the prevention of further fall. Consider this we are planning to do subgroup analysis with history >1 fall in the last year.<br><br>**Details of Statistical analysis is given in the protocol** .	
13.	The applicant has mentioned that they will obtain ethics approval, however, expected ethical issues of the study have not been identified. The consequences of deprescribing and ethical challenges need to be identified and addressed. ICMR's 12 principles may be referred to for this purpose	Thank you for the comment.<br>Ethical issues we may face during the conduct of trial are<br>**Therapeutic Misconception**: Participants may mistakenly believe that the tool-based intervention is the superior or preferred treatment option, potentially leading to therapeutic misconception. To mitigate this we will clearly communicate the purpose of the study, the randomization process, and the equipoise between the two study arms.<br>**Participant Vulnerability:** The study includes elderly population who are in FIDS. These may have multiple comorbidities, or are potentially more vulnerable due to cognitive impairment or limited decision-making capacity. Special care will be taken to protect their rights, autonomy, and well-being throughout the trial, including obtaining informed consent from a legally authorized representative when necessary.	44
14.	Recruitment strategy needs to be clearly identified.	Thank you for the suggestion.<br>The following Recruitment Strategy for Deprescribing Intervention Study in Elderly Patients on FRIDs will be implemented.<br>**Collaborating with Clinicians and Healthcare Providers**: We will work closely with clinicians and healthcare providers in the hospital, to identify eligible participants during their outpatient visits. This collaboration ensures that potential participants are well-informed about the study and referred to the research team for further evaluation.<br>**Targeting Elderly Patients on FRIDs**: Our recruitment strategy will specifically target elderly patients who are currently on FRIDs to ensure that the study participants meet the specific criteria relevant to the deprescribing intervention.	46-48

		**Engaging Caregivers and Family Members**: We will actively involve caregivers and family members who would be supporting elderly patients in the recruitment process by organizing education sessions specifically designed to address their concerns and provide guidance.<br>**Incentives:** To encourage enrollment and long-term engagement, we will offer incentives to participants. As part of the incentive package, participants will receive a maximum reimbursement of INR 500 per visit. This amount will cover their travel expenses and provide a small allowance for food during the study visits.<br>**Utilizing Electronic Health Records (EHRs)**: In the event that we are unable to obtain an adequate number of participants through the initial recruitment methods, we will leverage electronic health records systems. By analyzing patient data within these systems, we can identify suitable patients who are taking multiple medications, including FRIDs. These patients will be reach out through personalized communication channels, such as phone calls, to explain the deprescribing intervention and invite them to participate in the study.	

Budget comments

Sno	Reviewer comment	Response	Page no
1.	The reviewers commented that there may not be a need for a project technical officer and a nursing staff as their roles and responsibilities seem to be overlapping (absence of clear justifications by the applicant).	Thank you for the inputs. Please note that role of the project technical officer is distinct from that of the nursing staff. The project technical officer focuses on the technical and administrative aspects of the project, including data management, coordination with stakeholders at the site, ensuring compliance with regulatory requirements, and overseeing the implementation of the project protocols. These aspects will not within the purview of the nursing staff.<br>The nursing staff is involved in clinical assessments, monitoring adverse events, and ensuring the safety and well-being of the trial	52-57 Revised budget with justification

		participants.<br>However, as suggested we will try to manage with nursing staff	
2.	If required, one project technical officer may be recruited for the lead site for project coordination. These positions may be recruited only during months 10 to 35 in line with the gantt chart	Thank you for the suggestion.<br>As suggested, we will recruit a project technical officer for the lead site during the months indicated on the Gantt chart**.**	
3.	Compensation under recurring costs are not valid, especially since there is an insurance cover for the trial.	Thank you for the comments. It is true that we have insurance coverage for the trial participants, it is important to note that compensation under recurring costs serves a different purpose. Reimbursement is typically provided to trial participants to acknowledge their time, travel and any potential inconvenience they may experience while participating in the trial. It is not solely related to any adverse events or injuries that may occur during the trial.<br>Additionally, Compensation serves as a way to ensure participant engagement and retention, which is important for the success of this trial. It acts as an incentive for their continued involvement.	
4.	Budget containing insurance for the trial participants, lab tests and adverse event management could be removed/ revised. The intervention is not expected to have serious adverse events and the need for insurance, lab tests and adverse event management does not seem valid. Budget for data management can be increased	Thank you for the inputs.<br>**Insurance for Trial Participants**: While we anticipate that the intervention will not result in serious adverse events, it is essential to prioritize the safety and well-being of our trial participants. Providing insurance coverage ensures that participants have access to necessary medical care and financial protection in the unlikely event of any unforeseen complications. For Eg: fractures occurring due to fall which requires implants, cost of implants or procedural cost will be covered by Insurance.<br>**Incorporating lab tests** in this study helps to detect any subtle changes and identify potential adverse effects early on.<br>Although we don't expect serious adverse events in this study, it is important to be prepared for adverse event management.<br>As suggested we will remove the cost for managements of AEs alone.	
